# Gender awareness among general practitioners in France: a cross sectional study using the Nijmegen Gender Awareness in Medicine Scale (N-GAMS)

**DOI:** 10.1038/s41598-024-56396-5

**Published:** 2024-03-08

**Authors:** Perrine Goussault-Capmas, Henri Panjo, Nathalie Pelletier-Fleury

**Affiliations:** 1 CESP - Centre de Recherche en Epidémiologie et Santé des Populations, U1018 INSERM, Hôpital Paul Brousse, Université Paris Saclay, Université Versailles Saint Quentin, 16 Avenue Paul Vaillant Couturier, 94807 Villejuif Cedex, France; 2 Service Gynécologie Obstétrique, Hôpital de Bicêtre, 78 Avenue du Général Leclerc, 94275 Le Kremlin Bicêtre Cedex, France

**Keywords:** Health care, Signs and symptoms

## Abstract

Gender is a key determinant of health and healthcare use. The question of whether physicians are aware of gender issues is important to avoid gender bias in medical practice. This study aimed to validate the Nijmegen Gender Awareness in Medicine Scale (N-GAMS) in a representative population of French general practitioners (GPs) and to analyze their gender sensitivity and the presence of gender stereotypes among them. The N-GAMS, already validated in medical students, measures gender awareness through 3 subscores: gender sensitivity (GS) and gender-role ideology towards patients (GRIP) and doctors (GRID) (gender stereotypes). After translation into French, it was distributed to 900 GPs. The scale was validated through exploratory factor analysis (EFA). Psychometric properties were tested. Multivariate linear regressions were conducted to explore the associations between GPs’ characteristics and N-GAMS subscores. EFA identified 3 meaningful factors consistent with prior theory. Subscores exhibited good internal consistency. The main findings were that GRIP was significantly higher in older physicians, in male physicians, among those who less involved their patients in decisions, and those who were not training supervisors. For GRID, results were quite similar to those of GRIP. GS was significantly higher for physicians working in health centres or medical homes and for those with gynecological practices but lower when they less involved patients in medical decisions. This study suggests that it is necessary to teach gender issues not only in medical schools but also as part of continuing medical education.

## Introduction

Gender is a determinant of health inequalities either alone or in combination with other determinants such as socioeconomic status, age, and disability. While sex refers to the biological and physiological characteristics that differentiate men and women, gender refers to socially determined roles and behaviors, activities and attributes that a society considers appropriate for men and women (https://www.who.int/fr/news-room/fact-sheets/detail/gender).

Gender is an explanatory factor that is often accounted for in research on medical practices and health outcomes. It is frequently confused with sex, and the question of the interaction between gender and sex is often overlooked^1^. Gender is not a binary term. It includes an understanding that in many people, traits of masculinity and femininity coexist and are expressed to different degrees^2^. Currently, there is no universally accepted validated tool for measuring gender^1^. Canadian researchers have developed a gender index in a population of people with premature acute coronary events based on the gender concept developed by the Canadian Institutes for Health Research^3^ which comprises the four interrelated aspects of gender roles (e.g. childcare), identity (e.g., personality traits), relationships (e.g., social support) and gender social position (e.g., education level, personal income)^4^. Using this index, the authors showed that after adjusting for sex, female roles and personality traits were associated with a higher risk of recurrent acute coronary events^5^. This article demonstrates that these interrelated aspects of gender are determinants of patient care^2^.

The question of whether health professionals are aware of gender issues is important to avoid gender bias. Gender blindness and gender stereotypes are recognized as the main causes of gender bias^6^. Gender blindness is the failure to take gender into account whenever relevant. Gender stereotypes influence the interpretation of clinical signs and the management of conditions. For example, doctors are likely to interpret men's symptoms as organic and women's as psychosocial^7^. Thus, it is essential to take gender into account in medical practices to ensure quality and appropriate health care for men and women. This consideration, which is insufficient in routine practice, has led several European countries to integrate the gender issue into medical education to raise awareness among future doctors^8^. Gender awareness means that physicians acquire the knowledge and ability to recognize and integrate gender as a determinant of health and disease in their daily practice^9^. This is consistent with an awareness of the stereotypical beliefs about men’s and women’s behaviour, skills and needs that are incorrectly held in society. Since gender stereotypes can bias medical assessments, gender awareness involves reflecting on one’s own attitudes and preconceptions about men and women as well as patients and doctors^6^.

To our knowledge, no research has been conducted on gender awareness among physicians, particularly general practitioners (GPs). GPs are community-based practitioners who are in a leading position to address health inequalities^10^, including those related to gender bias. They are not only clinicians operating at all levels of care from prevention to palliative care, but they are also consulted very often. Across the EU, almost 3 in 10 (28.6%) males aged 15 years and over and more than one-third (36.3%) of females consulted a GP during the 4-week period leading up to the European health interview survey in 2019^11^. The difficulty may lie in measuring gender awareness. To our knowledge, only two scales have been developed and validated in the literature. The Gender Awareness Inventory-Veterans Affairs (GAI-VA) scale was developed to specifically assess health professionals' gender awareness for women veterans^12^ which hinders the more widespread use of this scale for both women and men in other care settings. The Nijmegen Gender Awareness in Medicine Scale (N-GAMS) measures medical students' gender awareness in terms of gender sensitivity and gender-role ideology towards patients and doctors (gender stereotypes). The Dutch team that validated the N-GAMS in English^9^ used it secondarily in collaboration with a Swedish team^8^. Cultural differences in the students’ responses to the questions were highlighted. This scale was adopted by a Swiss Romansh team who translated it and validated it in French. Their study concluded that medical students’ gender sensitivity seemed to improve throughout the medical curriculum, and that female students had fewer stereotypes towards patients than male students^13^. It has recently been used in Portugal^14^ and in Italy^15^. No validated scale is available in French for the population of GPs.

This study aimed to validate the N-GAMS in a representative population of French GPs and to analyse GPs' gender sensitivity and gender-role ideology towards patients and doctors.

## Materials and methods

### The original N-GAMS scale

The original N-GAMS scale^9^ is based on two attitudinal aspects of gender-awareness: gender sensitivity (GS) and gender role ideology which is assessed towards patients (GRIP) or doctors (GRID). GS is defined as the “ability to perceive existing gender differences, issues and inequalities and incorporate these into strategies and actions”^16^. GRIP and GRID refer to gender stereotypes towards patients and doctors, respectively. These three dimensions contain 14, 11 and 8 items that GPs assessed using a 5-point Likert scale (ranging from 1 “Strongly disagree” to 5 “Strongly agree”) (Table 1). Some items had reverse meaning; therefore, an adjustment of reverse scoring items was performed. The higher the item scores, the greater the gender sensitivity (GS) and gender stereotypes (GRIP and GRID).

**Table 1:** Nijmegen Gender Awareness in Medicine Scale (N-GAMS)^9^.

GS, Gender sensitivity (items scored in reverse_R)
GS1_R addressing differences between men and women creates inequity in health care
GS2 physicians’ knowledge of gender differences in illness and health increases quality of care*
GS3_R physicians should only address biological differences between men and women
GS4_R in non-sex-specific health disorders the sex/gender of the patient is irrelevant
GS5_R a physician should confine as much as possible to biomedical aspects of health complaints of men and women
GS6_R physicians do not need to know what happens in the lives of men and women to be able to deliver medical care*
GS7_R differences between male and female physicians are too small to be relevant
GS8_R especially because men and women are different, physicians should treat everybody the same
GS9_R physicians who address gender differences are not dealing with the important issues
GS10_R in communicating with patients it does not matter to a physician whether the patients are men or women
GS11_R in communicating with patients it does not matter whether the physician is a man or a woman
GS12_R differences between male and female patients are so small that physicians can hardly take them into account
GS13 for effective treatment, physicians should address gender differences in etiology and consequences of disease
GS14_R it is not necessary to consider gender differences in presentation of complaints
GRIP, Gender role ideology towards patients
GRIP1 male patients better understand physicians’ measures than female patients
GRIP2 female patients compared to male patients have unreasonable expectations of physicians
GRIP3 women more frequently than men want to discuss problems with physicians that do not belong in the consultation room
GRIP4 women expect too much emotional support from physicians
GRIP5 male patients are less demanding than female patients
GRIP6 women are larger consumers of health care than is actually needed
GRIP7 men do not go to a physician for harmless health problems
GRIP8 medically unexplained symptoms develop in women because they lament too much about their health
GRIP9 female patients complain about their health because they need more attention than male patients
GRIP10 it is easier to find causes of health complaints in men because men communicate in a direct way
GRIP11 men appeal to health care more often with problems they should have prevented
GRID, Gender role ideology towards doctors
GRID1 male physicians put too much emphasis on technical aspects of medicine compared to female physicians
GRID2 female physicians extend their consultations too much compared to male physicians
GRID3 male physicians are more efficient than female physicians
GRID4 female physicians are more empathic than male physicians
GRID5 female physicians needlessly take into account how a patient experiences disease
GRID6 male physicians are better able to deal with the work than female physicians
GRID7 female physicians are too emotionally involved with their patients
GRID8 compared to female physicians, male physicians are too hurried in their consultations

### GP recruitment

In total 3530 GPs were invited to participate by a polling company specialized in health surveys (https://www.b3tsi.com). The recruitment of participants was conducted through e-mail and by phone.

### Validation of the N-GAMS scale

#### Translation-retranslation of the N-GAMS

The English version of the original N-GAMS was translated into French by the study team and then back-translated into English by a professional translator. The two English versions (initial and retranslated versions) were compared, item by item, by all participants and disagreements on the French version was discussed and solved (vf N-GAMS).

#### Exploratory factor analysis (EFA)

Given the potential uncertainty surrounding the structure of the N-GAMS scale for French practising GPs, we chose to perform an exploratory rather than confirmatory factor analysis^17^.

##### Appropriateness of the Spearman correlation matrix for EFA

First, we checked the appropriateness of the Spearman correlation matrix for EFA using (i) visual examination of the correlations (% of significant correlations and of correlations $\ge$ 0.30)^18,19^, (ii) Bartlett’s test of sphericity which tests the overall significance of the correlation^20^, and (iii) the Kaiser–Meyer–Olkin measure of sampling adequacy (MSA) for the entire correlation matrix (overall MSA) and for the 33 individual items (item specific MSA). MSA values above 0.50 indicate appropriateness for performing factor analysis on the overall set of items or specific items^21^. The overall MSA quantifies the degree of intercorrelations among items, and an item-specific MSA quantifies the item’s correlation with the other items in the analysis.

##### Determination of the number of factors to retain for rotation

Common factor analysis (iterated principal axis extraction) was used because the purpose of this study was to uncover the latent structure underlying these 33 measured items^22^. To determine the number of factors to retain, we first referred to the original validation study^9^ (a priori criterion), which suggested that the scale had three dimensions. Since its development, the N-GAMS has been used several times, and researchers have extracted the same number of factors^8,13–15^. We also used a visual scree test^23^ supplemented by a modified latent root criterion^18^. The classical latent root criterion, also known as the Kaiser rule, is a stopping rule where all factors with eigenvalues (latent roots) greater than 1 are retained, whereas the modified version recommends that only the factors with eigenvalues greater than the average of the item-specific MSA are considered significant, which is argued to be more appropriate in a common factor analysis^18^. This criterion is usually considered reliable when the number of variables is between 20 and 50, which was the case in this study (33) and item-specific MSA above 0.40^18^.

##### Model acceptability

For both theoretical and empirical reasons, it was assumed that factors would be correlated^24,25^. Thus, an oblique Promax rotation with a k value of 4 was selected^26^. An oblique rotation honours the ubiquity of intercorrelations among social science variables^25^ and “Promax rotation is almost always a good choice”^27^. The threshold for salience for loadings was set at 0.30 to meet the minimal level for interpretation of structure^18^, that is, variables with approximately 9% (factor loading squared) of their variance explained by the factor.

Following guidelines for model acceptability, (i) three salient item loadings (pattern coefficients) are necessary to form a factor^24^, (ii) the root mean squared residual (RMSR), which is a measure of overall residual misfit values, must be ≤ 0.08^28^, (iii) the proportion of nonredundant residual correlations greater than the absolute value of 0.10 should be small ^29^, and (iv) the results across alternative extraction (iterated principal axis, ordinary least squares, weighted least squares, minimum residual) and rotation methods (promax, oblimin) must be robust.

#### Item reliability

Item reliability was assessed through two diagnostic measures of internal consistency. First, a Cronbach’s alpha coefficient was calculated for each sub-scale. It should be at least 0.70. Second, for each subscale, item-rest score correlations between the items and the rest scores of the subscale (i.e., the score computed from the items of the dimension deleting that item) were computed, using Spearman correlations. The absolute value of the item-rest correlations should be above 0.1. Absolute values between 0.1 and 0.3 are considered “fair”, while those above 0.3 are deemed “good”^20^.

#### Bivariate correlations between the subscores

Bivariate correlations between the dimensions were also conducted. *p* values were adjusted for multiple comparisons with the Holm method.

### Analysis of French GPs’ gender sensitivity and stereotypes

#### Descriptive analysis

We first performed a descriptive analysis of GPs’ characteristics and scores on the NGAMS by dimension. We used the so called « dummy » coding for all categorical variables, and age has been segmented into 4 classes. For a GP, the score in a dimension (called the subscore) was obtained by averaging the observed values of the items in the dimension. Mean scores (95% confidence interval) for the NGAMS by dimension were calculated.

#### Linear regressions

The relationship between GPs’ characteristics and subscores was analysed through univariate linear regressions. GPs’ characteristics with a *p* value at 0.2 or less were included in multiple linear regression models. A value < 0.05 was considered statistically significant.

### Ethics statements

This study was approved by the the Inserm Ethics Evaluation Committee (Comité d’évaluation éthique de l’Inserm), under the 22-895. We confirm that informed consent to participate was obtained from all of the participants in the study and that all methods were performed in accordance with the relevant guidelines and regulations.

## Results

A total of 3530 GPs were contacted (3454 received a link via the internet, and 76 were contacted by phone). Of these, 2479 refused to answer (2425 and 54, respectively). The response rate was 30%. Of the 1051 respondents, 151 had incomplete questionnaires. The study population was 900 GPs.

### Validation of the N-GAMS scale

#### Translation-retranslation of the N-GAMS

The vf N-GAMS is available upon request.

#### Exploratory factor analysis (EFA)

##### Appropriateness of the data for EFA

Inspection of the correlation matrix reveals that 366 of the 528 correlations (69%) were significant at the 0.01 level, and 168 of the 528 correlations (32%) were ≥ 0.30. Bartlett’s test of sphericity rejected the hypothesis that the correlation matrix was an identity matrix (Bartlett χ^2^ (528) = 12,708.279, *p* < 0.001). Therefore the correlation matrix contained statistically significant correlations. The overall MSA value fell in the meritorious range (above 0.80) with a value of 0.938. Examination of the item-specific MSA values for each variable yielded a range from 0.68 to 0.97, with 31 out of 33 MSA values above 0.80, and an average of 0.91. Taken together, these measures indicate that our data were appropriate for EFA.

##### Determination of the number of factors to retain for rotation

**Figure 1:**
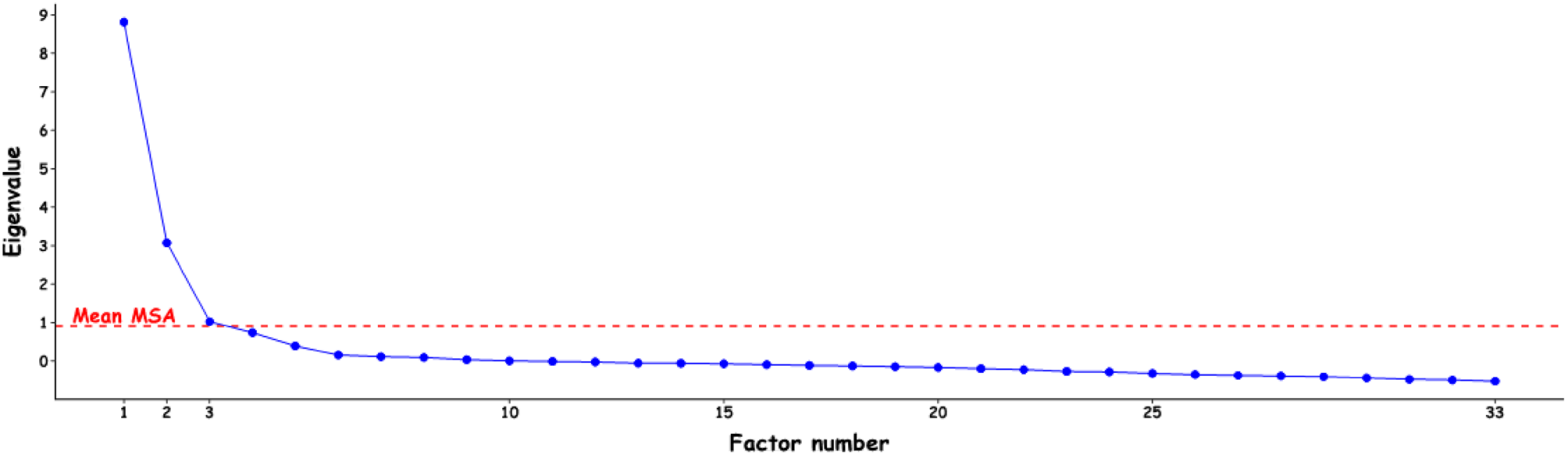
Scree test and modified latent root criteria.

The average of the item-specific MSA was 0.91. Three factors had eigenvalues greater than the average of the MSA. Figure 1 indicates that both latent root and scree test criteria suggest retaining 3 factors, confirming the a priori criteria.

##### Model acceptability

Table 2 shows the results of the EFA. The measured items were distributed across the three factors as predicted by prior theory and the structure was interpretable and theoretically meaningful. One item, GRIP 11, was under the limit for saliency (0.22); three items did not load sufficiently on their own dimension GRID 3 (0.19), GRID 5 (0.20) and GRID 6 (0.14), but on the GRIP dimension (with loadings > 0.30). These items were removed. Two items (GS2 and GS13) had loadings at the limit of saliency (0.29), but we retained them as Hair et al.^18^ suggested that for a sample size of n = 350, the threshold for loading saliency is 0.30. The sample size of our study was n = 900; therefore, a loading of 0.29 clearly meets the minimal level for the interpretation of structure. In summary, 4 loadings (14%) met the minimal level for the interpretation of structure (0.29–0.40), 15 loadings (52%) were practically significant (0.40–0.70), and 10 loadings (34%) were indicative of well-defined structure (> 0.70). Altogether, the three latent factors accounted for 41.52% of the total variance of the original data. The first factor (GRIP) explained 25.99% of the total variance, the second factor (GS) explained 11.49%, and the third factor (GRID) explained 4.04% of the total variance.

**Table 2:** Exploratory factor analysis of the N-GAMS (n = 900 general practitionners).

Item	Factor loadings		
	GS	GRIP	GRID
GS, Gender sensitivity (items scored in reverse R)			
GS12R Differences between male and female patients are so small that physicians can hardly take them into account	**0.65**	− 0.07	− 0.02
GS4R In non-sex-specific health disorders the sex/gender of the patient is irrelevant	**0.60**	0.10	− 0.03
GS14R It is not necessary to consider gender differences in presentation of complaints	**0.57**	0.09	− 0.07
GS1R Addressing differences between men and women creates inequity in health care	**0.55**	− 0.03	− 0.13
GS3R Physicians should only address biological differences between men and women	**0.53**	− 0.11	0.00
GS7R Differences between male and female physicians are too small to be relevant	**0.52**	− 0.03	0.13
GS9R Physicians who address gender differences are not dealing with the important issues	**0.52**	− 0.07	− 0.06
GS10R In communicating with patients it does not matter to a physician whether the patients are men or women	**0.52**	0.10	0.09
GS5R A physician should confine as much as possible to biomedical aspects of health complaints of men and women	**0.49**	− 0.11	− 0.04
GS8R Especially because men and women are different, physicians should treat everybody the same	**0.42**	0.01	− 0.06
GS11R In communicating with patients it does not matter whether the physician is a man or a woman	**0.40**	0.08	0.04
GS6R Physicians do not need to know what happens in the lives of men and women to be able to deliver medical care	**0.34**	− 0.15	− 0.02
GS13 For effective treatment, physicians should address gender differences in etiology and consequences of disease	**0.29**	0.14	0.04
GS2 Physicians' knowledge of gender differences in illness and health increases quality of care	**0.29**	− 0.01	0.05
GRIP, Gender role ideology towards patients			
GRIP8 Medically unexplained symptoms develop in women because they lament too much about their health	− 0.01	**0.88**	− 0.09
GRIP9 Female patients complain about their health because they need more attention than male patients	0.02	**0.84**	− 0.06
GRIP2 Female patients compared to male patients have unreasonable expectations of physicians	− 0.01	**0.84**	− 0.06
GRIP6 Women are larger consumers of health care than is actually needed	0.00	**0.81**	− 0.05
GRIP5 Male patients are less demanding than female patients	0.01	**0.77**	− 0.01
GRIP1 Male patients better understand physicians' measures than female patients	− 0.04	**0.77**	0.00
GRIP10 It is easier to find causes of health complaints in men because men communicate in a direct way	− 0.02	**0.72**	− 0.04
GRIP7 Men do not go to a physician for harmless health problems	− 0.01	**0.71**	− 0.06
GRIP4 Women expect too much emotional support from physicians	0.02	**0.62**	0.17
GRIP3 Women more frequently than men want to discuss problems with physicians that do not belong in the consultation room	0.05	**0.60**	0.11
GRID, Gender role ideology towards doctors			
GRID8 Compared to female physicians, male physicians are too hurried in their consultations	0.01	− 0.05	**0.81**
GRID4 Female physicians are more empathic than male physicians	0.02	− 0.13	**0.79**
GRID2 Female physicians extend their consultations too much compared to male physicians	− 0.04	0.11	**0.65**
GRID1 Male physicians put too much emphasis on technical aspects of medicine compared to female physicians	0.01	0.14	**0.64**
GRID7 Female physicians are too emotionally involved with their patients	− 0.04	0.22	**0.58**
Items removed from the exploratory factor analysis			
GRIP11 Men appeal to health care more often with problems they should have prevented			
GRID3 Male physicians are more efficient than female physicians			
GRID5 Female physicians needlessly take into account how a patient experiences disease			
GRID6 Male physicians are better able to deal with the work than female physicians			

*N-GAMS* Nijmegen Gender Awareness in Medicine Scale.

Significant loadings are bold.

RMSR was low at 0.036, and only 4 (0.99%) of the residuals were above the absolute value of 0.10, indicating no presence of another factor.

The results across alternative extraction and rotation methods were robust (i.e., gave similar solutions). Factor analysis on different subsamples (gender, age) also gave similar solutions.

#### Item reliability

The Cronbach’s α values were αGRIP = 0.925 [0.917, 0.932] for the GRIP subscale, αGS = 0.806 [0.787, 0.824] for the GS subscale, and αGRID = 0.849 [0.833, 0.864] for the GRID subscale.

Twelve GS items out of 14 (86%), 100% of GRIP items and 100% of GRID items had an item-rest correlation greater than 0.30.

#### Bivariate correlations between the subscores

The GRIP score and GRID score were positively correlated ($r =$ 0.611; *p* < 0.001), no significant correlation was found between the GS score and GRID score ($r =$  − 0.027; *p* = 0.426), and no significant correlation was found between the GS score and GRIP score ($r =$  − 0.066; *p* = 0.096).

### Analysis of French GPs’ gender sensitivity and stereotypes

#### Descriptive analysis

**Figure 2:**
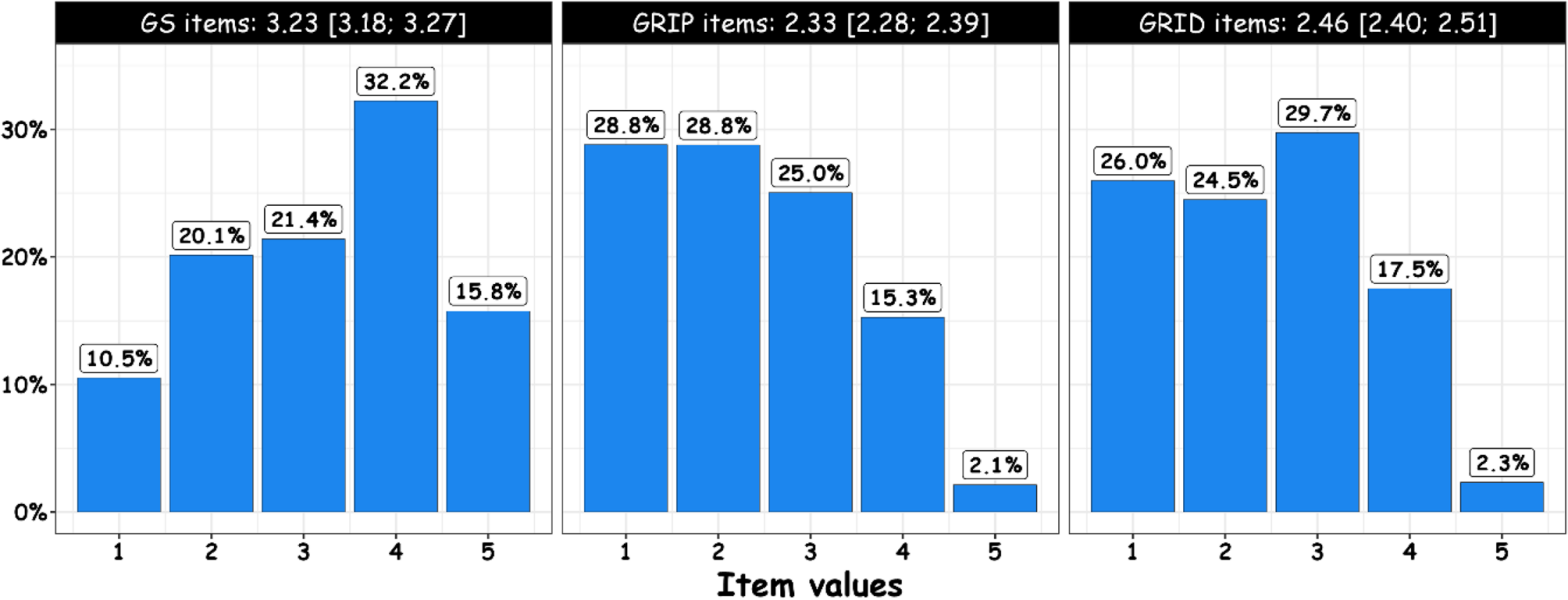
Distribution of item values and mean scores for the NGAMS by dimension.

The 900 recruited GPs were representative in terms of age, sex and urban/rural practice of the general population of GPs in France. Their characteristics are summarized in Table 3. Scores for the NGAMS by dimension are presented in Fig. 2. The mean GS, GRIP, and GRID scores were 3.23 (3.18–3.27), 2.33 (2.28–2.39) and 2.46 (2.40–2.51), respectively. GS items were scored 3 or more in 69% of cases, which suggests medium to high gender sensitivity, while GRIP and GRID items were scored 4 or 5 in 17.4 and 19.8% of cases, respectively reflecting significant gender stereotypes towards patients and doctors in this population. The descriptive statistics of the N-GAMS items for all study populations and by sex are summarized in Tables 4 and 5.

**Table 3:** General practionners’ characteristics (n = 900).

Characteristics	Total
	% (n)
Demographics	
Sex	
Female	41.2 (371)
Male	58.8 (529)
Age (years)	
27–40	13.2 (119)
41–55	38.0 (342)
56–65	37.0 (333)
66–79	11.8 (106)
Professional characteristics	
Type of exercise	
Private practice only	88.2 (794)
Other	11.8 (106)
Practice setting	
Rural	21.3 (192)
Semi urban	27.1 (244)
Urban	51.6 (464)
Training supervisor	
No	75.1 (676)
Yes	24.9 (224)
Number of consultations per week	
≤ 115	43.6 (392)
> 115	56.4 (508)
Number of years of practice	
2–17	26.3 (237)
18–25	25.8 (232)
26–32	22.1 (199)
33–55	25.8 (232)
Gynecological practice	
No	50.4 (454)
Yes	49.6 (446)
Patients involved in medical decisions	
Not at all/little/moderately	21.4 (193)
Quite a lot	46.0 (414)
Systematically	32.6 (293)
Informed on gender issues	
No	77.4 (697)
Yes	22.6 (203)
Personal characteristics	
Living in couple	
No	11.0 (99)
Yes	89.0 (801)
Spouse occupation	
Farmers/craftsmen/shopkeepers	6.7 (60)
Executives and higher intellectual professions	52.4 (472)
Intermediate professions, technicians, foremen	12.6 (113)
Employes/workers	6.7 (60)
Unemployed	10.7 (96)
Not in couple	11.0 (99)
Have children	
No	13.4 (121)
Yes	86.6 (779)
Perceived health status	
Very bad/bad	13.0 (117)
Good	57.3 (516)
Very good/excellent	29.7 (267)
Have chronic disease	
No	72.4 (652)
Yes	27.6 (248)

**Table 4:** Descriptive statistics of the N-GAMS (n = 900 general practitionners).

Item	Descriptive statistics		
	Mean (SE)	Skewness	Kurtosis
GS, Gender sensitivity (items scored in reverse R)			
GS12R Differences between male and female patients are so small that physicians can hardly take them into account	3.39 (0.04)	− 0.41	− 0.62
GS4R In non-sex-specific health disorders the sex/gender of the patient is irrelevant	2.83 (0.04)	0.09	− 1.10
GS14R It is not necessary to consider gender differences in presentation of complaints	3.11 (0.04)	− 0.12	− 1.06
GS1R Addressing differences between men and women creates inequity in health care	3.49 (0.04)	− 0.44	− 0.97
GS3R Physicians should only address biological differences between men and women	3.33 (0.04)	− 0.32	− 0.92
GS7R Differences between male and female physicians are too small to be relevant	2.97 (0.04)	0.02	− 0.96
GS9R Physicians who address gender differences are not dealing with the important issues	3.59 (0.04)	− 0.54	− 0.60
GS10R In communicating with patients it does not matter to a physician whether the patients are men or women	2.71 (0.04)	0.23	− 1.11
GS5R A physician should confine as much as possible to biomedical aspects of health complaints of men and women	3.45 (0.04)	− 0.44	− 0.92
GS8R Especially because men and women are different, physicians should treat everybody the same	3.16 (0.04)	− 0.19	− 0.73
GS11R In communicating with patients it does not matter whether the physician is a man or a woman	2.68 (0.04)	0.23	− 0.95
GS6R Physicians do not need to know what happens in the lives of men and women to be able to deliver medical care	3.88 (0.03)	− 1.02	0.51
GS13 For effective treatment, physicians should address gender differences in etiology and consequences of disease	3.09 (0.04)	− 0.35	− 0.79
GS2 Physicians' knowledge of gender differences in illness and health increases quality of care	3.47 (0.04)	− 0.75	− 0.03
GRIP, Gender role ideology towards patients			
GRIP8 Medically unexplained symptoms develop in women because they lament too much about their health	2.23 (0.04)	0.46	− 0.81
GRIP9 Female patients complain about their health because they need more attention than male patients	2.25 (0.04)	0.40	− 0.89
GRIP2 Female patients compared to male patients have unreasonable expectations of physicians	2.08 (0.03)	0.58	− 0.53
GRIP6 Women are larger consumers of health care than is actually needed	2.45 (0.04)	0.30	− 0.85
GRIP5 Male patients are less demanding than female patients	2.34 (0.04)	0.37	− 0.91
GRIP1 Male patients better understand physicians' measures than female patients	1.93 (0.03)	0.54	− 0.74
GRIP10 It is easier to find causes of health complaints in men because men communicate in a direct way	2.21 (0.03)	0.53	− 0.44
GRIP7 Men do not go to a physician for harmless health problems	2.55 (0.04)	0.19	− 1.06
GRIP4 Women expect too much emotional support from physicians	2.56 (0.04)	0.08	− 0.94
GRIP3 Women more frequently than men want to discuss problems with physicians that do not belong in the consultation room	2.71 (0.04)	− 0.02	− 1.14
GRID, Gender role ideology towards doctors			
GRID8 Compared to female physicians, male physicians are too hurried in their consultations	2.51 (0.04)	0.13	− 1.00
GRID4 Female physicians are more empathic than male physicians	2.64 (0.04)	0.03	− 1.07
GRID2 Female physicians extend their consultations too much compared to male physicians	2.45 (0.04)	0.18	− 1.05
GRID1 Male physicians put too much emphasis on technical aspects of medicine compared to female physicians	2.31 (0.03)	0.22	− 0.90
GRID7 Female physicians are too emotionally involved with their patients	2.38 (0.04)	0.24	− 0.96
Items removed from the exploratory factor analysis			
GRIP11 Men appeal to health care more often with problems they should have prevented	2.98 (0.04)	− 0.38	− 1.02
GRID3 Male physicians are more efficient than female physicians	1.77 (0.03)	0.96	− 0.02
GRID5 Female physicians needlessly take into account how a patient experiences disease	1.90 (0.03)	0.71	− 0.33
GRID6 Male physicians are better able to deal with the work than female physicians	1.99 (0.04)	0.71	− 0.62

*N-GAMS* Nijmegen Gender Awareness in Medicine Scale.

**Table 5:** Descriptive statistics of the N-GAMS by sex (n = 900 general practitionners).

Items	Female N = 371	Male N = 529	*p* value
	Mean (SE)	Mean (SE)	
GS, Gender sensitivity (items scored in reverse_R)			
GS1_R Addressing differences between men and women creates inequity in health care	3.55 (0.065)	3.45 (0.056)	0.242
GS2 Physicians’ knowledge of gender differences in illness and health increases quality of care	3.53 (0.055)	3.43 (0.047)	0.176
GS3_R Physicians should only address biological differences between men and women	3.40 (0.062)	3.28 (0.054)	0.158
GS4_R In non-sex-specific health disorders the sex/gender of the patient is irrelevant	2.73 (0.065)	2.90 (0.054)	0.048
GS5_R A physician should confine as much as possible to biomedical aspects of health complaints of men and women	3.64 (0.063)	3.31 (0.055)	< 0.001
GS6_R Physicians do not need to know what happens in the lives of men and women to be able to deliver medical care	3.96 (0.048)	3.83 (0.048)	0.051
GS7_R Differences between male and female physicians are too small to be relevant	3.08 (0.059)	2.90 (0.051)	0.017
GS8_R Especially because men and women are different, physicians should treat everybody the same	3.13 (0.061)	3.19 (0.050)	0.520
GS9_R. Physicians who address gender differences are not dealing with the important issues	3.68 (0.061)	3.53 (0.052)	0.077
GS10_R In communicating with patients it does not matter to a physician whether the patients are men or women	2.65 (0.067)	2.75 (0.055)	0.256
GS11_R In communicating with patients it does not matter whether the physician is a man or a woman	2.63 (0.062)	2.71 (0.053)	0.318
GS12_R For effective treatment, physicians should address gender differences in etiology and consequences of disease	3.43 (0.054)	3.36 (0.049)	0.352
GS13 For effective treatment, physicians should address gender differences in etiology and consequences of disease	3.04 (0.059)	3.12 (0.050)	0.261
GS14_R It is not necessary to consider gender differences in presentation of complaints	3.06 (0.062)	3.14 (0.054)	0.301
GRIP, Gender role ideology towards patients			
GRIP1 Male patients better understand physicians’ measures than female patients	1.78 (0.047)	2.03 (0.041)	< 0.001
GRIP2 Female patients compared to male patients have unreasonable expectations of physicians	1.96 (0.052)	2.17 (0.045)	0.002
GRIP3 Women more frequently than men want to discuss problems with physicians	2.76 (0.064)	2.67 (0.050)	0.249
GRIP4 Women expect too much emotional support from physicians	2.55 (0.058)	2.56 (0.047)	0.946
GRIP5 Male patients are less demanding than female patients	2.17 (0.054)	2.46 (0.050)	< 0.001
GRIP6 Women are larger consumers of health care than is actually needed	2.37 (0.058)	2.51 (0.049)	0.057
GRIP7 Men do not go to a physician for harmless health problems	2.46 (0.060)	2.62 (0.051)	0.044
GRIP8 Medically unexplained symptoms develop in women because they lament too much about their health	2.09 (0.055)	2.32 (0.048)	0.001
GRIP9 Female patients complain about their health because they need more attention than male patients	2.09 (0.055)	2.37 (0.047)	< 0.001
GRIP10 It is easier to find causes of health complaints in men because men communicate in a direct way	2.06 (0.048)	2.31 (0.046)	< 0.001
GRID, Gender role ideology towards doctors			
GRID1 Male physicians put too much emphasis on technical aspects of medicine compared to female physicians	2.37 (0.057)	2.26 (0.044)	0.111
GRID2 Female physicians extend their consultations too much compared to male physicians	2.44 (0.061)	2.46 (0.048)	0.889
GRID4 Female physicians are more empathic than male physicians	3.00 (0.060)	2.39 (0.049)	< 0.001
GRID7 Female physicians are too emotionally involved with their patients	2.42 (0.061)	2.35 (0.046)	0.373
GRID8 Compared to female physicians, male physicians are too hurried in their consultations	2.62 (0.058)	2.43 (0.048)	0.009

*N-GAMS* Nijmegen Gender Awareness in Medicine Scale.

#### Linear regressions

The results of the univariate linear regressions are presented in Table 6. The following variables sex, age, type of exercise, training supervisor, number of years of practice, gynecological practice, patients involved in medical decision which had a *p* value less than 0.2 in at least one of the three models were included in multiple linear regression models. In the multivariate analysis (Table 7), gender sensitivity was lower for doctors who did not involve their patients at all or involved them moderately in medical decisions than for those who involved them a lot (*p* = 0.007) and was higher for doctors working in settings other than private practices (*p* = 0.049) and for those with gynaecological practice (*p* = 0.036). Gender stereotypes towards patients were significantly more important the older the doctors were, with an increasing gradient of GRIP scores (*p* < 0.001). They were also higher among male doctors than among female doctors (*p* = 0.023), among those who did not involve or moderately involved their patients in decisions (*p* = 0.01) and among those who were not training supervisors (*p* = 0.05). Gender stereotypes towards doctors were also associated with age (*p* < 0.001) and were higher among those who did not involve or moderately involved their patients in decisions (*p* = 0.014). They were lower among male doctors than among female doctors (*p* < 0.001).

**Table 6:** Relationship between general practitionners’ characteristics (n = 900) and N-GAMS subscores through univariate linear regression.

Characteristics	Score GS		Score GRIP		Score GRID	
	Observed mean	Coefficient [CI 95%]	Mean	Coefficient [CI 95%]	Mean	Coefficient [CI 95%]
Sex						
Female (371)	3.25	Ref	2.23	Ref	2.57	Ref
Male (529)	3.21	− 0.04 [− 0.13, 0.04]	2.40	0.17 [0.06, 0.28]**	2.38	− 0.20 [− 0.31, − 0.08]**
Age (years)						
27–40 (119)	3.22	Ref	2.09	Ref	2.19	Ref
41–55 (342)	3.20	− 0.02 [− 0.15, 0.11]	2.26	0.17 [0.00, 0.35]	2.42	0.23 [0.05, 0.42]
56–65 (333)	3.25	0.02 [− 0.11, 0.16]	2.42	0.33 [0.16, 0.51]**	2.54	0.35 [0.17, 0.53]**
66–79 (106)	3.24	0.01 [− 0.15, 0.18]	2.54	0.45 [0.23, 0.67]**	2.59	0.40 [0.17, 0.63]**
Type of exercise						
Private practice only (794)	3.21	Ref	2.33	Ref	2.46	Ref
Other (106)	3.36	0.15 [0.02, 0.28]*	2.32	− 0.01 [− 0.18, 0.16]	2.46	0.01 [− 0.17, 0.19]
Practice setting						
Rural (192)	3.20	Ref	2.29	Ref	2.45	Ref
Semi urban (244)	3.26	0.06 [− 0.06, 0.18]	2.30	0.01 [− 0.15, 0.17]	2.44	− 0.01 [− 0.18, 0.15]
Urban (464)	3.22	0.02 [− 0.08, 0.13]	2.37	0.08 [− 0.07, 0.22]	2.47	0.02 [− 0.13, 0.17]
Training supervisor						
No (676)	3.23	Ref	2.39	Ref	2.50	Ref
Yes (224)	3.21	− 0.02 [− 0.12, 0.08]	2.16	− 0.23 [− 0.35, − 0.10]**	2.34	− 0.16 [− 0.29, − 0.03]*
Number of consultations per week						
≤ 115 (392)	3.26	Ref	2.29	Ref	2.48	Ref
> 115 (508)	3.20	− 0.07 [− 0.15, 0.02]	2.36	0.07 [− 0.04, 0.18]	2.44	− 0.04 [− 0.15, 0.08]
Number of years of practice						
2–17 (237)	3.23	Ref	2.19	Ref	2.33	Ref
18–25 (232)	3.17	− 0.07 [− 0.18, 0.05]	2.25	0.06 [− 0.09, 0.21]	2.43	0.10 [− 0.06, 0.25]
26–32 (199)	3.29	0.05 [− 0.06, 0.17]	2.46	0.26 [0.11, 0.42]**	2.49	0.16 [0.00, 0.33]
33–55 (232)	3.23	− 0.01 [− 0.12, 0.11]	2.45	0.25 [0.10, 0.41]**	2.59	0.25 [0.10, 0.41]**
Gynecological practice						
No (454)	3.17	Ref	2.37	Ref	2.46	Ref
Yes (446)	3.28	0.10 [0.02, 0.19]*	2.30	− 0.07 [− 0.18, 0.04]	2.46	0.00 [− 0.12, 0.11]
Patients involved in medical decisions						
Systematically (293)	3.31	Ref	2.13	Ref	2.29	Ref
Quite a lot (414)	3.23	− 0.07 [− 0.17, 0.02]	2.36	0.23 [0.10, 0.35]**	2.48	0.19 [0.06, 0.32]**
Not at all/little/moderately (193)	3.09	− 0.22 [− 0.33, − 0.11]*	2.57	0.44 [0.29, 0.59]**	2.66	0.38 [0.22, 0.53]**
Informed on gender issues						
No (697)	3.21	Ref	2.33	Ref	2.47	Ref
Yes (203)	3.29	0.08 [− 0.01, 0.18]	2.34	0.01 [− 0.12, 0.14]	2.42	− 0.05 [− 0.19, 0.09]
Living in couple						
No (99)	3.25	Ref	2.40	Ref	2.59	Ref
Yes (801)	3.22	− 0.03 [− 0.16, 0.11]	2.32	− 0.08 [− 0.25, 0.10]	2.44	− 0.15 [− 0.34, 0.03]
Spouse occupation						
Farmers/craftsmen/shopkeepers (60)	3.32	Ref	2.3	Ref	2.4	Ref
Executives and higher intellectual professions (472)	3.20	− 0.12 [− 0.29, 0.05]	2.27	− 0.03 [− 0.25, 0.20]	2.43	− 0.01 [− 0.25, 0.23]
Intermediate professions, technicians, foremen (113)	3.30	− 0.02 [− 0.22, 0.17]	2.40	0.10 [− 0.16, 0.36]	2.48	0.03 [− 0.24, 0.31]
Employees/workers (60)	3.20	− 0.12 [− 0.35, 0.10]	2.35	0.05 [− 0.25, 0.35]	2.38	− 0.06 [− 0.38, 0.25]
Unemployed (96)	3.17	− 0.15 [− 0.35, 0.05]	2.48	0.18 [− 0.09, 0.45]	2.48	0.03 [− 0.25, 0.32]
Not in couple (99)	3.25	− 0.08 [− 0.28, 0.13]	2.40	0.10 [− 0.17, 0.37]	2.59	0.15 [− 0.13, 0.43]
Have children						
No (121)	3.26	Ref	2.39	Ref	2.48	Ref
Yes (779)	3.22	− 0.04 [− 0.16, 0.08]	2.32	− 0.07 [− 0.23, 0.09]	2.45	− 0.03 [− 0.20, 0.14]
Perceived health status						
Very bad/bad (117)	3.31	Ref	2.36	Ref	2.48	Ref
Good (516)	3.22	− 0.08 [− 0.21, 0.04]	2.32	− 0.04 [− 0.21, 0.13]	2.47	− 0.01 [− 0.19, 0.17]
Very good/excellent (267)	3.19	− 0.11 [− 0.25, 0.02]	2.34	− 0.02 [− 0.20, 0.16]	2.43	− 0.05 [− 0.24, 0.14]
Have chronic disease						
No (652)	3.21	Ref	2.34	Ref	2.45	Ref
Yes (248)	3.26	0.04 [− 0.05, 0.14]	2.32	− 0.02 [− 0.14, 0.11]	2.48	0.04 [− 0.09, 0.16]

*N-GAMS* Nijmegen Gender Awareness in Medicine Scale.

*p < 0.05; **p < 0.01.

**Table 7:** Relationship between general practionionners’ characteristics (n = 900) and N-GAMS subscores through multivariate linear regression.

Characteristics	Score GS		Score GRIP		Score GRID	
	Coef [CI95%]	*p* val	Coef [CI95%]	*p* val	Coef [CI95%]	*p* val
Constant	3.13 [2.97, 3.29]	< 0.001	1.95 [1.74, 2.15]	< 0.001	2.32 [2.10, 2.54]	< 0.001
Age (years)						
27–40 (119)	Ref		Ref		Ref	
41–55 (342)	− 0.02 [− 0.15, 0.11]	0.803	0.20 [0.02, 0.37]	0.025	0.24 [0.06, 0.42]	0.009
56–65 (333)	0.03 [− 0.10, 0.17]	0.624	0.30 [0.12, 0.47]	< 0.001	0.36 [0.18, 0.54]	< 0.001
66–79 (106)	0.00 [− 0.16, 0.17]	0.968	0.39 [0.18, 0.61]	< 0.001	0.42 [0.19, 0.65]	< 0.001
Sex						
Female (371)	Ref		Ref		Ref	
Male (529)	− 0.01 [− 0.09, 0.08]	0.904	0.13 [0.02, 0.25]	0.023	− 0.25 [− 0.37, − 0.13]	< 0.001
Patients involved in medical decisions						
Systematically (293)	0.06 [− 0.03, 0.16]	0.200	− 0.22 [− 0.34, − 0.09]	< 0.001	− 0.19 [− 0.32, − 0.06]	0.004
Quite a lot (414)	Ref		Ref		Ref	
Not at all/little/moderately (193)	− 0.15 [− 0.26, − 0.04]	0.007	0.18 [0.04, 0.32]	0.010	0.18 [0.04, 0.33]	0.014
Training supervisor						
No (676)	0.05 [− 0.05, 0.15]	0.305	0.13 [0.00, 0.26]	0.050	0.06 [− 0.08, 0.20]	0.381
Yes (224)	Ref		Ref		Ref	
Type of exercise						
Private practice only (794)	Ref		Ref		Ref	
Other (106)	0.13 [0.00, 0.26]	0.049	0.07 [− 0.10, 0.24]	0.414	0.04 [− 0.13, 0.22]	0.641
Gynecological practice						
No (454)	Ref		Ref		Ref	
Yes (446)	0.09 [0.01, 0.18]	0.036	0.00 [− 0.11, 0.12]	0.964	− 0.04 [− 0.16, 0.08]	0.516

*N-GAMS* Nijmegen Gender Awareness in Medicine Scale (N-GAMS).

## Discussion

We validated the NGAMS in a population of French GPs and used this scale to analyse GPs’ gender awareness in this population. We showed that GS was positively associated with care practices (involving patients more in decisions, and working in health centres (centres de santé) or medical homes (maisons de santé pluriprofessionnelles) compared with working in private practice), and practising gynaecology), while GRIP, although also positively associated with care practices (not at all/little/moderately involving patients in the decision, and not being training supervisors), was also associated with GPs’ sociodemographics (being male, older). For GRID, results were quite similar to those of GRIP, except that male doctors had fewer gender stereotypes towards doctors than female doctors.

An EFA of the N-GAMS instrument's items was used to identify the latent structure underpinning the instrument's 33 items. Although^9^ reported using principal component analysis, we chose to use principal axis factoring instead as it appears to be a particularly suitable means of extracting latent factors based on the shared variance of the variables^22^. The final set of 29 accepted items produced a 3-dimensional structure of gender awareness: GS (14 items), GRIP (10 items), and GRID (5 items). These three subscales had satisfactory internal consistency (alpha > 0.80). We found a quite high association between the GRID and GRIP subscales, similar to other authors suggesting a common ground for GRIP and GRID. However, the data did not show any evidence of correlation between GS and the other two subscales, which also supported earlier findings^9,14^. This indicates that these are separate aspects of the attitudinal component of gender awareness, which may need to be targeted and addressed independently in interventions^14^.

The N-GAMS has never been validated among GPs. Therefore, there are no comparative measures of gender awareness in this population. It should be noted that the sample of 900 GPs we surveyed was representative in terms of age, gender, rural/urban practice and type of practice (private surgery/others) of GPs in France^30^.

With regard to GS, the result we found of an independent association of three variables describing care practices, i.e., involving patients in decisions, practising gynaecology, and working in health centres or medical homes, with GS is original. Its originality lies both in the fact that it has never been shown before and in the interpretation that could be drawn from it, namely, that these associations could be mediated by patient-centred practices. Following Lindsay et al., GS is a key component of patient-centred care^31^. Applying a gender-sensitive perspective in patient-centred care requires that healthcare providers can perceive existing sex and gender differences, issues and inequalities and incorporate these into strategies and actions. The patient-centred approach applied to ambulatory care^32^ implies respect for the patient's values, preferences, and expressed needs, information and education, access to care, emotional support to relieve fear and anxiety, involvement of family and friends, continuity and secure transition between health care settings, physical comfort, and coordination of care. In this framework, shared decision making (i.e., when the patient is involved to reach the optimal decision) is seen as the pinnacle of patient-centred care^33^. Moreover, prior experiences, notably with patient-centred care medical homes in the US, show that these healthcare organizations should allow better monitoring of the care provided to ensure that it corresponds to the best standards, increase the possibilities of interaction and communication with patients, to encourage their participation in the care process, and better coordinate the intervention of the various stakeholders in the care process^32^. This is the equivalent of the multiprofessional health centres in France, whose number is increasing. The GPs who work in these healthcare organizations are part of this process of providing patient-centred rather than disease-centred care, even if the organizational practices needed to implement and adopt patient-centred care remain incomplete^34^. Finally, regarding OB/GYN practices, developmental issues such as menstruation, contraceptive initiation, pregnancy, childbirth, and menopause that are addressed by GPs can be transitional periods of difficulty such as unwanted pregnancy, infertility, pregnancy loss, chronic illness and pain, mood and sleep disorders, interpersonal trauma, and poverty, and may serve as opportunities to address the complexity of patients’ needs^35^, reflecting the interest of these physicians in the patient-centred approach^32^.

With regard to GRIP, and the association between being a male doctor and having more gender stereotypes, in four of the NGAMS validation studies conducted among medical students^8,9,13,15^, male medical students held stronger gender stereotypes than female medical students. These findings may explain, at least in part, the effects of patients’ and physicians’ gender discordance on patient management observed in general practice. Indeed, several studies conducted in general practice have shown that the management of female patients by male doctors (as opposed to female doctors) may be detrimental in the area of cancer screening for women in the USA^36^, health promotion (advice on physical exercise and weight loss) and the management of hypertension in France^37,38^, or physicians’ perception of uncertainty about a diagnosis and hidden agenda beyond the reason(s) for the visit in New Zealand^39^. Our results also show that, all other things being equal, gender stereotypes towards patients increase with the age of the GP, with a very clear gradient, with a mean GRIP score from 2.09 (between 27 and 40 years old) to 2.54 (between 66 and 79 years old). Looking at the validation studies of the N-GAMS among medical students, the results of the effect of age on gender stereotypes towards patients vary from one study to another. In multivariate linear models, stereotypes towards patients decreased linearly with students getting older in Switzerland (ranging from 18 to 32 years old) (coefficient − 0.03, *p* = 0.035) (Rrustemi et al.) and in Sweden (coefficient − 0.189, *p* < 0.001) (Andersson et al.). In contrast, older students expressed more stereotypical thinking about patients in Italy (coefficient 0.04, *p* value = 0.012)^15^. No significant relationships were found in Portugal^14^. As Bert et al. suggested, Swedish and Swiss medical students reduced their stereotypes probably due to a good theoretical and practical teaching system^15^. It is not clear from our study whether stereotypes towards patients are the result of an ageing effect or a cohort effect. Indeed, given that the oldest GPs we interviewed belonged to the 1943–1956 birth cohort (aged 66–79 years in 2022) while the youngest belonged to the 1982–1995 cohort (aged 27–40 years in 2022), it can be assumed that growing up during such distinctive historical times influenced gender stereotypes towards patients^40^. There are no data in the literature to support this hypothesis. We would need longitudinal data to disentangle these ageing and cohort effects. Additionally, gender stereotypes towards patients increased when GPs did not their patients at all, little or moderately in medical decisions, with a mean GRIP score ranging from 2.13 (patients systematically involved) to 2.57. This result is consistent with those in the literature. Within the framework of an ecological model of communication in medical encounters, Street^41^ stated that gender-based perceptions and stereotypes can play a prominent role in medical encounters, although, we still know very little about the scope of these beliefs and their impact. Sandhu et al.^42^ further claimed that non concordant gender dyads may be characterized by perceived differences in power, status, dominance, gender stereotypes, and attitudes towards the other sex that may lead to higher levels of tension and a lower communication quality. In a study performed in primary care where the consultations were videotaped, the provision of patient-centred care (measured by coding the videotapes using the Davis Observation Code) was shown to be influenced by gender concordance: female concordant dyads were associated with a greater amount of patient-centred care^43^. Finally, not being a training supervisor was associated with more gender stereotypes towards patients with a mean GRIP score ranging from 2.16 (being a training supervisor) to 2.39. This result, which to our knowledge, has not previously been reported, could be explained in several ways. GPs who choose to be supervisors are different from others, particularly in terms of gender, professional practice and training: women are overrepresented, as are practising in multiprofessional health centres and having additional training^44^; in other words this profile of doctors may have fewer gender stereotypes towards patients. Furthermore, being a supervisor implies a triangular doctor patient relationship that can have numerous potential benefits, not only for the training of residents. The presence of residents introduces a new perspective on medical situations and practice as well as on a given doctor patient relationship^45^.

Regarding GRID, our results show that, all other things being equal, gender stereotypes towards doctors increase with the age of the GP with a very clear gradient, with a mean GRID score from 2.19 (between 27 and 40 years old) to 2.59 (between 66 and 79 years old). As with GRIP, a cohort effect can probably partly explain this result. The GP profession has been undergoing feminization for several decades. In France, the proportion of female GPs almost doubled between 2000 and 2021 alone, rising from 24 to 43%^30^. Young male and female doctors today have shared faculty benches and practice in an environment consisting almost equally of male and female doctors, which makes them less prone to gender stereotyping towards their colleagues compared to older doctors. However, all things being equal, the mean GRID score was significantly higher for female doctors (2.57) than for male doctors (2.38). In particular, female doctors were considered more empathetic with patients than men doctors, and male doctors were believed to be too rushed during consultations. Women doctors (due to feedback from patients or discussions between colleagues) may have integrated the differences in practice between male and female doctors that have been demonstrated^46^.

## Conclusion

This study is the first to measure gender awareness in a population of GPs. All the results discussed above allow us to conclude that it seems necessary to teach gender issues in medical schools. This is already the case in some countries, such as Sweden and Switzerland. We may perhaps go further by suggesting that gender be taught as part of continuing medical education.

## Data Availability

Data and the French version of the N-GAMS are available upon request from Henri Panjo (henri.panjo@inserm.fr).
